# Nivel de conocimiento sobre enfermedad periodontal en estudiantes universitarios del área de salud y otras áreas. Un estudio transversal

**DOI:** 10.21142/2523-2754-1304-2025-259

**Published:** 2025-11-08

**Authors:** Alexandra Jacqueline Aquino Valverde, Datfne Milagros Barrientos Sánchez, César Alonso Ponce Lozano

**Affiliations:** 1 Facultad de Estomatología, Universidad Científica del Sur. Lima, Perú. aaquinovalverde@gmail.com datfnebsanz@gmail.com Universidad Científica del Sur Facultad de Estomatología Universidad Científica del Sur Lima Peru aaquinovalverde@gmail.com datfnebsanz@gmail.com; 2 División de Periodoncia e Implantología, Facultad de Estomatología, Universidad Científica del Sur. Lima, Perú. cponcel@cientifica.edu.pe Universidad Científica del Sur División de Periodoncia e Implantología Facultad de Estomatología Universidad Científica del Sur Lima Peru cponcel@cientifica.edu.pe

**Keywords:** medicina periodontal, conocimientos, actitudes y prácticas de salud, encuestas y cuestionarios, periodontal medicine, knowledge, health attitudes and practices, surveys, and questionnaires

## Abstract

**Introducción:**

La enfermedad periodontal (EP), que incluye patologías como la gingivitis y la periodontitis, afecta entre el 20% y el 50% de la población mundial, y es una de las principales causas de pérdida dentaria. Esta condición compromete funciones como la masticación, la estética y la calidad de vida.

**Objetivo:**

Determinar el nivel de conocimiento sobre enfermedad periodontal en estudiantes universitarios de carreras del área de salud y no salud en una universidad privada del Perú.

**Metodología:**

Se realizó un estudio transversal comparativo con una muestra por conveniencia de 388 estudiantes (192 del área de salud y 196 del área no salud). Se utilizó un cuestionario autoadministrado de 15 ítems, que evaluó conocimientos sobre salud periodontal (7 ítems), higiene oral (4 ítems) y datos sociodemográficos (4 ítems). La validez del instrumento fue respaldada por un índice KMO de 0,65 y una prueba de esfericidad de Bartlett significativa (p < 0,001). La confiabilidad se determinó mediante el coeficiente omega de McDonald (0,615). El análisis estadístico incluyó pruebas de chi-cuadrado y regresión lineal múltiple, con un nivel de significancia de p < 0,05.

**Resultados:**

El 53,6% de los participantes reportó cepillarse tres veces al día, siendo esta práctica más común entre los estudiantes del área no salud (p < 0,001). En cuanto al conocimiento sobre EP, el 72,7% presentó un nivel bajo, sin diferencias significativas según sexo (p = 0,39) ni área académica (p = 0,29). Solo el 1% alcanzó un nivel alto de conocimiento.

**Conclusión:**

Los estudiantes universitarios evaluados presentaron un bajo nivel de conocimiento sobre enfermedad periodontal, lo que evidencia la necesidad de integrar contenidos de salud bucal en la formación universitaria de manera transversal y multidisciplinaria.

## Abstract (en)

**Introduction:**

Periodontal disease (PD), which includes conditions such as gingivitis and periodontitis, affects between 20% and 50% of the global population and is one of the leading causes of tooth loss. This condition compromises functions such as mastication, aesthetics, and overall quality of life.

**Objective:**

To determine the level of knowledge about periodontal disease among university students from health and non-health programs at a private university in Peru.

**Methodology:**

A comparative cross-sectional study was conducted with a convenience sample of 388 students (192 from health-related programs and 196 from non-health-related programs). A self-administered 15-item questionnaire was used to assess knowledge in three areas: periodontal health (7 items), oral hygiene (4 items), and sociodemographic data (4 items). The validity of the instrument was supported by a KMO index of 0.65 and a significant Bartlett's sphericity test (p<0.001). Reliability was determined using McDonald’s omega coefficient (0.615). Statistical analysis included Chi-square tests and multiple linear regression, with a significance level set at p<0.05.

**Results:**

A total of 53.6% of participants reported brushing their teeth three times a day, with this practice being more common among non-health students (p<0.001). Regarding knowledge of PD, 72.7% demonstrated a low level of knowledge, with no significant differences by sex (p=0.39) or academic area (p=0.29). Only 1% exhibited a high level of knowledge.

**Conclusion:**

The evaluated university students showed a low level of knowledge about periodontal disease, highlighting the need to integrate oral health content into university curricula in a cross-cutting and multidisciplinary manner.

## INTRODUCCIÓN

La salud es un estado de bienestar mental, social y físico que va más allá de la ausencia de una patología ^(1, 2^). Según la Organización Mundial de la Salud (OMS), la salud periodontal está asociada a la ausencia de inflamación periodontal ^(3, 4^). La enfermedad periodontal (EP) es una enfermedad osteolítica crónica que conduce a la pérdida del diente, se refiere a las condiciones que conducen a la degeneración de los tejidos de soporte del diente, clasificadas por estadios y grados ^(5, 6^). La EP con una prevalencia de entre el 20% y el 50% en todo el mundo, siendo las más comunes la gingivitis y la periodontitis, es una de las principales causas de pérdida de dientes que afecta la masticación, la estética, la pérdida de confianza y la calidad de vida ^(7, 8^). La EP es multifactorial y se asocia con enfermedades sistémicas como diabetes, enfermedades cardiovasculares, obesidad y problemas durante el embarazo ^(9^). La limitada conciencia y educación en torno a la salud periodontal en los países subdesarrollados constituye un factor determinante en la prevalencia y progresión de las enfermedades periodontales ^(10, 11^).

La EP provoca disminución del periodonto que conlleva pérdida de adherencias periodontales y pérdida de dientes, lo que afecta la calidad de vida, pérdida de función masticatoria y aumenta los costos del tratamiento ^(12^). Dadas las condiciones clínicas en nuestro país y la alta prevalencia de la EP, mantener la salud periodontal pública es de suma importancia y limita las vías económicas para prevenir estas enfermedades ^(13^).

Hay antecedentes en la literatura científica que evalúan el nivel de conocimiento sobre la EP entre estudiantes de otras etnias, y se encontró en estos estudiantes niveles bajos de conocimiento, lo que proyecta consecuencias negativas en el futuro. Sin embargo, también hay estudios en los que se hallaron niveles aceptables de conocimiento, debido a que estos estudiantes en su estructura universitaria sí contemplan cursos de salud periodontal ^(14-18^). Por otro lado, existen pocos estudios en Latinoamérica que aborden los conocimientos en salud periodontal, a pesar del aumento en el número de enfermedades periodontales ^(19^), por lo que es necesario saber si las personas no relacionadas con la odontología tienen los conocimientos básicos para prevenirla ^(20^). La EP afecta la cavidad oral y la salud general del individuo; por ello, se le reconoce como un determinante de la morbilidad de la enfermedad sistémica ^(21^).

A diferencia de estudios previos centrados exclusivamente en estudiantes de carreras de salud, este estudio aporta un enfoque novedoso al comparar el nivel de conocimiento sobre enfermedad periodontal entre estudiantes universitarios de distintas áreas académicas dentro de una misma institución. Esta comparación permite identificar brechas en la alfabetización sobre salud bucal en la población universitaria general, y con ello genera evidencia local útil para sustentar la necesidad de integrar contenidos básicos de salud bucal en los planes de estudio de manera transversal y multidisciplinaria. En tal sentido, es importante determinar el nivel de conocimientos sobre enfermedad periodontal en estudiantes universitarios del área de la salud y otras áreas en la ciudad de Lima (Perú), mediante un estudio transversal.

## MATERIALES Y MÉTODOS

### Diseño del estudio y ética

El estudio es de tipo comparativo-transversal y fue aprobado por el Comité Institucional de Ética en Investigación de la Universidad Científica del Sur (N.° 323-CIEI-CIENTÍFICA-2023). Asimismo, fue-ron respetados los principios éticos de la Declaración de Helsinki mediante la entrega de consentimiento informado a los participantes para que accedan al estudio, y se codificaron los datos recogidos mediante generación automática para garantizar su confidencialidad. Al finalizar la aplicación del cuestionario, se proporcionó a cada participante un afiche educativo con información sobre medidas de prevención en salud periodontal. Esta acción tuvo como finalidad promover la educación en salud bucal y contribuir a la sensibilización de los estudiantes con respecto a la importancia del cuidado periodontal, lo cual cumple el principio ético de beneficencia en la investigación.

### Muestra y criterio de selección

Se empleó un muestreo no probabilístico por conveniencia, conformado por un total de 388 estudiantes mayores de 18 años pertenecientes a una universidad privada ubicada en Lima, Perú. De este grupo, 192 estudiantes cursaban carreras del área de la salud, mientras que 196 provenían de carreras no relacionadas con dicho campo. La elección de esta técnica de muestreo respondió a la necesidad de acceder a una población específica dentro del ámbito universitario. Asimismo, su uso se justificó por razones prácticas, tales como la facilidad de contacto con los participantes, su disposición para colaborar en la investigación, así como por limitaciones de tipo logístico, temporal y de recursos disponibles.

Se incluyeron en el estudio aquellos estudiantes universitarios que otorgaron su consentimiento informado para participar voluntariamente en la investigación y que se encontraban cursando entre el quinto y décimo ciclo académico. La muestra estuvo conformada por estudiantes de carreras del área de la salud, como Medicina Humana, Enfermería y Obstetricia, así como de carreras no vinculadas con el área de salud, como Ingeniería Económica, Ingeniería Empresarial, Derecho, Artes Escénicas, Economía y Finanzas, Ingeniería Ambiental, y Arquitectura y Urbanismo Ambiental. Además, se excluyeron del estudio aquellos estudiantes que no completaron adecuadamente el cuestionario, quienes contaban con estudios previos en odontología, así como aquellos que participaron en la fase piloto de validación del instrumento.

### Adaptación, validación y confiabilidad del cuestionario de conocimientos

Se extrajo el Cuestionario de Conocimiento de Salud Periodontal (CSP) de un estudio previo ^(^²²) y se tradujo profesionalmente al español. Por otro lado, cuatro especialistas en periodoncia e implantología, con más de 10 años de experiencia, evaluaron el cuestionario en función de los criterios de coherencia, claridad, suficiencia y relevancia, utilizando una escala del 1 (no cumple) al 4 (alto nivel). El coeficiente V de Aiken valoró los resultados en 0,92. Asimismo, para evaluar la validación por constructo y la confiabilidad, se aplicó una encuesta piloto a 70 estudiantes de carreras distintas a la muestra principal (35 de salud y 35 de no salud). El análisis factorial exploratorio confirmó una validación aceptable (Kayser-Meyer-Olkin: 0,655; test de Bartlett: < 0,001). Se utilizó el coeficiente omega de McDonald para evaluar la confiabilidad interna del instrumento, y se obtuvo un valor de 0,615. Aunque este valor se sitúa por debajo del umbral convencional de 0,70 propuesto para estudios confirmatorios, se considera aceptable dentro del contexto de investigaciones exploratorias, cuya principal finalidad es identificar patrones preliminares y orientar el desarrollo de futuras herramientas de medición más robustas. A diferencia del alfa de Cronbach, el coeficiente omega proporciona una estimación más precisa y realista de la consistencia interna, pues considera los pesos factoriales individuales de cada ítem, lo que permite una evaluación más adecuada del constructo en contextos donde las cargas factoriales son heterogéneas.

### Descripción y aplicación del cuestionario

El instrumento utilizado fue un cuestionario estructurado compuesto por 15 preguntas distribuidas en tres secciones: características sociodemográficas (4 ítems), hábitos de higiene oral (4 ítems) y conocimientos sobre salud periodontal (7 ítems). La sección sociodemográfica incluyó variables como sexo, edad, carrera profesional y ciclo académico. Las preguntas sobre higiene oral abordaron aspectos como la frecuencia del cepillado dental, el uso de elementos auxiliares de limpieza bucal y la frecuencia de visitas al odontólogo. La sección correspondiente al conocimiento sobre salud periodontal fue la única en la que se aplicó un sistema de puntuación de 0 a 7, en función del número de respuestas correctas. Posteriormente, los puntajes obtenidos se categorizaron en tres niveles: bajo (0-3 puntos), medio (4-5 puntos) y alto (6-7 puntos). Esta sección evaluó conocimientos relacionados con los signos y síntomas, factores asociados y medidas de prevención de las enfermedades periodontales. La encuesta fue aplicada de manera presencial en las instalaciones de la universidad, utilizando un formulario elaborado en la plataforma Google Forms.

### Análisis estadístico

Se recopilaron los datos mediante encuestas virtuales y se registraron en una hoja de cálculo de Microsoft Excel, donde fueron codificados y depurados. Posteriormente, se exportaron al programa STATA v17 para su análisis estadístico.

La información se procesó en tablas porcentuales y se representó a través de gráficos de barras y de dispersión. Para analizar la asociación entre variables, se aplicó la prueba de chi-cuadrado, considerando la variable “Conocimientos” de manera categórica (bajo y alto nivel). Además, se identificaron los factores que influyen en un bajo nivel de conocimientos mediante un modelo de regresión lineal múltiple. En todos los análisis, se estableció un umbral de significancia de 0,05.

## RESULTADOS

En la tabla 1 se presentan las características sociodemográficas de los 388 estudiantes universitarios, con predominio del sexo femenino (69,1%). La distribución por área académica fue equilibrada entre estudiantes de carreras de salud (49,5%) y no salud (50,5%), lo que permitió una comparación equitativa conforme al objetivo del estudio. La mayoría se encontraba en ciclos intermedios o avanzados, y destacó el séptimo ciclo como el más representado (30,1%). Las carreras con mayor participación femenina fueron Obstetricia, Medicina y Enfermería. Asimismo, se incluyeron estudiantes de diversas disciplinas no relacionadas con la salud, como Arquitectura, Derecho, Economía e Ingeniería, lo que aportó una muestra heterogénea y adecuada para describir las características sociodemográficas en relación con el conocimiento sobre enfermedad periodontal.

La tabla 2 presenta la distribución de las prácticas de higiene bucal según el sexo y la condición de salud bucal de los participantes. Esta muestra diferencias significativas en la frecuencia del cepillado diario según el área académica (p < 0,001), siendo más frecuente entre estudiantes de carreras no relacionadas con la salud. Sin embargo, no se encontraron diferencias significativas en esta práctica entre sexos (p = 0,73). El uso de hilo dental y enjuague bucal no presentó diferencias estadísticamente significativas ni por sexo ni por área académica, aunque su uso fue ligeramente mayor en mujeres. En cuanto a la visita al odontólogo, se identificó una diferencia significativa por sexo (p = 0,01), con mayor frecuencia en mujeres, pero sin diferencias relevantes entre estudiantes de salud y no salud (p = 0,54).

La tabla 3 muestra la distribución de respuestas sobre conocimientos acerca de la enfermedad periodontal (EP) según sexo y tipo de carrera (salud vs. no salud). Presenta resultados variados entre los participantes. Solo el 44,8% identificó correctamente la placa bacteriana como factor inicial de la EP, y el 38,1% reconoció el sangrado gingival como signo principal, sin diferencias estadísticamente significativas por sexo ni área académica. El 72,7% señaló correctamente el método más efectivo de prevención, aunque también sin diferencias significativas. Sin embargo, el 52,3% presentó una concepción errónea sobre el uso del enjuague bucal para eliminar el mal aliento. Se encontró una diferencia significativa por sexo en el reconocimiento del vínculo entre la EP y las enfermedades cardíacas (p = 0,03), con mayor proporción de respuestas correctas en mujeres. Respecto a la relación con la diabetes, el 71,6% respondió correctamente, sin diferencias significativas. Finalmente, se halló una diferencia significativa por área académica en la relación entre EP y tabaquismo (p = 0,04), siendo más acertadas las respuestas del grupo de salud.

En la tabla 4 se presenta la distribución del nivel de conocimientos sobre enfermedad periodontal (EP) según el sexo y el área académica de los participantes. La mayoría de los estudiantes (72,7%) presentó un nivel bajo de conocimiento sobre enfermedad periodontal, seguido por un 26,3% con nivel medio y solo un 1% con nivel alto. No se encontraron diferencias estadísticamente significativas según sexo (p = 0,39) ni área académica (p = 0,29), aunque se observó una ligera tendencia a mejores niveles de conocimiento en mujeres y en estudiantes del área de salud. En general, los resultados reflejan un conocimiento insuficiente sobre la EP en la población estudiada, independientemente del sexo o la carrera.

## DISCUSIÓN

La enfermedad periodontal (EP) es una de las patologías orales crónicas más prevalentes a nivel mundial, con una afectación estimada entre el 20% y el 50% de la población. A pesar de su alta incidencia y su impacto directo en la salud bucal, funciones masticatorias, estética y calidad de vida, la EP sigue siendo subestimada tanto por la población general como por estudiantes universitarios, incluidos los vinculados con las ciencias de la salud. Diversas investigaciones han evidenciado niveles insuficientes de conocimiento sobre esta patología, incluso en futuros profesionales del sector salud, lo que plantea preocupaciones sobre su capacidad para identificar, prevenir o educar sobre estas enfermedades en su práctica futura. En este contexto, el presente estudio tuvo como propósito determinar el nivel de conocimientos sobre enfermedad periodontal en estudiantes universitarios del área de la salud y otras áreas de Lima, Perú, mediante un estudio transversal.

Los resultados obtenidos en este estudio evidencian que, a pesar de la formación académica en el área de salud, los estudiantes universitarios presentan un nivel de conocimiento insuficiente sobre la enfermedad periodontal (EP), sin que el sexo o la carrera de estudio influyan significativamente en este aspecto. La mayoría de los participantes (72,7%) mostró un nivel bajo de conocimiento sobre la EP, con una ligera tendencia hacia un mayor porcentaje de mujeres con nivel medio de conocimiento (18,3%) en comparación con los varones (7,9%). Sin embargo, estas diferencias no fueron estadísticamente significativas (p = 0,39). De manera similar, al analizar la relación con el área académica, tanto los estudiantes del área de salud (75%) como los de otras áreas (70,4%) mostraron predominantemente un nivel bajo de conocimientos, sin diferencias significativas (p = 0,29).

Estos hallazgos coinciden con estudios previos que han reportado niveles insuficientes de conocimiento sobre la EP entre estudiantes universitarios, independientemente de su sexo o área de estudio. Por ejemplo, un estudio realizado en una universidad privada de Lima, Perú, encontró que el 29,5% de los estudiantes de ciencias de la salud presentaron un nivel bajo de conocimiento sobre salud periodontal, a pesar de su formación en el área de salud ^(23^). Asimismo, una investigación realizada en Kuwait ^(24^) reveló que, aunque las estudiantes de ciencias de la salud mostraron un mayor conocimiento sobre salud bucal, las diferencias entre sexos no fueron estadísticamente significativas, lo que sugiere que la brecha en el conocimiento puede no estar necesariamente condicionada por el género.

Además, la frecuencia de prácticas preventivas como el cepillado dental, el uso de hilo dental o enjuague bucal, y las visitas al dentista, aunque reportadas por un número considerable de participantes, no mostraron asociación significativa con el sexo ni con la carrera estudiada, a excepción del cepillado, que fue mayor en estudiantes del área de no salud (p < 0,001), y la visita al odontólogo, significativamente más frecuente en mujeres (p = 0,01). Estos resultados son consistentes con estudios recientes que señalan que la frecuencia y calidad de los hábitos de higiene bucal entre estudiantes universitarios no siempre se correlacionan con su área de estudio, incluso en el caso de disciplinas relacionadas con la salud ^(25, 26^).

Estos resultados refuerzan la necesidad de incorporar estrategias educativas más eficaces sobre salud bucal y enfermedades periodontales dentro de los programas académicos, incluso en carreras del área de salud. Un estudio ^(27^) ha evidenciado que intervenciones educativas dirigidas pueden mejorar significativamente el conocimiento y la actitud hacia la salud periodontal en poblaciones universitarias.

Los hallazgos de este estudio, que revelan un bajo nivel de conocimiento sobre enfermedad periodontal en estudiantes universitarios incluso en aquellos del área de la salud, tienen implicancias clínicas relevantes. Una formación deficiente en salud bucal durante la etapa universitaria puede traducirse en profesionales con escasa capacidad para reconocer signos tempranos de enfermedad periodontal o para promover prácticas preventivas entre sus pacientes o comunidades. En el caso específico de carreras como Medicina, Enfermería u Obstetricia, donde existe contacto directo con pacientes, la falta de conocimientos básicos sobre la EP podría limitar la detección oportuna de manifestaciones orales vinculadas a condiciones sistémicas. Este estudio evidencia la necesidad de incorporar contenidos fundamentales de salud bucal en los currículos de formación universitaria de manera transversal, a fin de fortalecer la atención integral del paciente, fomentar la prevención interdisciplinaria y mejorar los resultados clínicos en salud oral desde un enfoque más amplio y colaborativo.

### Limitaciones

A pesar de los aportes que este estudio ofrece para comprender el nivel de conocimientos y prácticas de higiene bucal en estudiantes universitarios, se deben considerar algunas limitaciones. En primer lugar, se trató de un estudio transversal, lo que impide establecer relaciones causales entre las variables analizadas. En segundo lugar, la muestra fue no probabilística y estuvo limitada a una sola universidad, lo que restringe la generalización de los resultados a otras poblaciones universitarias. Además, el uso de un cuestionario autoadministrado puede haber estado sujeto a sesgos de autopercepción o deseabilidad social, especialmente en preguntas relacionadas con prácticas de higiene personal. Finalmente, si bien se logró una distribución equilibrada entre estudiantes de salud y no salud, no se consideraron diferencias curriculares específicas dentro de cada carrera que pudieran haber influido en los conocimientos sobre la EP.

En futuras investigaciones, sería recomendable ampliar la muestra a varias universidades; incluir variables adicionales, como antecedentes familiares de enfermedad periodontal; y emplear métodos mixtos que combinen cuestionarios con entrevistas o evaluaciones clínicas, para obtener una comprensión más profunda y validada del problema.

## CONCLUSIÓN

El estudio transversal determinó que la mayoría de los estudiantes, tanto de salud como de otras áreas, presentan un nivel bajo de conocimiento sobre enfermedad periodontal (72,7%). No se encontraron diferencias significativas según el área académica ni el sexo. Solo el 1% alcanzó un nivel alto. Estos hallazgos evidencian una brecha formativa en salud bucal a nivel universitario.

## Fuente de financiamiento:

Autofinanciado
